# Soft Skills: An Important Asset Acquired from Organizing Regional Student Group Activities

**DOI:** 10.1371/journal.pcbi.1003708

**Published:** 2014-07-03

**Authors:** Jeroen de Ridder, Pieter Meysman, Olugbenga Oluwagbemi, Thomas Abeel

**Affiliations:** 1 Delft Bioinformatics Lab, Delft University of Technology, Delft, The Netherlands; 2 Biomedical Informatics Research Center Antwerp (biomina), University of Antwerp/Antwerp University Hospital, Antwerp, Belgium; 3 Department of Mathematics and Computer Sciences, University of Antwerp, Antwerp, Belgium; 4 Department of Computer and Information Sciences, School of Natural and Applied Sciences, Covenant University, Ota, Nigeria, West Africa; 5 Broad Institute of MIT and Harvard, Cambridge, Massachusetts, United States of America; 6 VIB Department of Plant Systems Biology, Ghent University, Ghent, Belgium; Princeton University, United States of America

## Abstract

Contributing to a student organization, such as the International Society for Computational Biology Student Council (ISCB-SC) and its Regional Student Group (RSG) program, takes time and energy. Both are scarce commodities, especially when you are trying to find your place in the world of computational biology as a graduate student. It comes as no surprise that organizing ISCB-SC-related activities sometimes interferes with day-to-day research and shakes up your priority list. However, we unanimously agree that the rewards, both in the short as well as the long term, make the time spent on these extracurricular activities more than worth it. In this article, we will explain what makes this so worthwhile: soft skills.

## Introduction

The term soft skill refers to one of the many different aspects of the social behavior required to become successful at your job, be it a career in academia or in industry. These attributes can include communications skills and coaching and leadership abilities, as well as personal qualities such as friendliness, empathy, and optimism [1]. Undoubtedly, they are crucial assets on the road to becoming a successful scientist.

Many of these skills are hard to acquire by just reading a book. Instead, they can only be learned through practice. For young researchers in the field of computational biology, an interesting opportunity to improve their soft skillset is to become an active member of the International Society for Computational Biology Student Council (ISCB-SC) or one of its Regional Student Groups (RSGs).

## Communication Skills

Most will agree that, within an organization, smooth internal communication and clear external communication are of vital importance. Despite many books and theories on this topic, very few universities feature an extensive list of courses on successful communication for the more scientific disciplines. Perhaps it is expected that you learn this on the job. In our experience, this is certainly the case when you volunteer for a task within the Student Council.

Because the RSGs are a virtual organization (i.e., they typically do not have an office), internal communication greatly depends on the effective use of online collaboration tools. These tools, such as Skype, Google Docs, and Basecamp, are increasing in quality and effectiveness. As a result, keeping many people in the loop and involving volunteers from all continents has become much easier. Nevertheless, it remains a real challenge to efficiently plan meetings in six time zones or have effective ten-way Skype calls. This requires specific communication skills that can be readily acquired by on-the-job training from more experienced volunteers.

*“If you make the effort of reflecting on your communication skills and consciously try to improve them, you will soon notice interaction with your peers becomes more and more effective.”*—Jeroen de Ridder (Student Council chair in 2010 and former board member of RSG-Netherlands)

Effective external communication is essential for an RSG because it is crucial to achieving its main mission: reaching out to students in the field of computational biology. The most important challenge is to engage the scientific community and inform them about our activities and events. This requires the skill to write captivating advertisements that clearly communicate both the utility as well as the fun of an event. This is only useful if sent through the proper channels. For this purpose, it is beneficial to have a strong network at your disposal (see also: Networking skills).

*“When there are many people involved in a certain project, there are an equal number of different opinions and ideas floating around during a meeting. How do you put forward your idea and how do you convince the others of it? One strategy is to make sure you collect the ideas that are somewhat similar to yours and merge them with your proposal. In my experience, your idea becomes even better, plus you get a few people on your side since they will recognize their own ideas reflected in yours.”*—Jeroen de Ridder

## Organizational Skills

RSG-organized events frequently require organization of a multitude of matters, such as the budget, sponsors, scientific content, time schedule, speakers, food, and coffee. For the responsible volunteer(s), this demands strong organizational skills. These skills are readily acquired when you operate in a team in which the responsibilities are shared among its members. For this reason, almost all RSG-organized events are organized as a team effort.

*“When working with a group of people and as part of a large organization, it is essential to set clear goals and objectives to base your future decisions on. Every RSG is required to define these goals at start-up and to reevaluate them annually. This way you clearly define what the main tasks are that you want to accomplish and how you plan to achieve them. This is a vital skill to have before undertaking any larger project. I have found myself setting goals and objectives for every new research project after my experience with the RSG and have become more efficient because of it.”*—Pieter Meysman (president of RSG-Belgium, 2011 to 2012).

Since many RSGs consist of an international group of students and organize joint events, volunteers frequently have to collaborate with people of very diverse backgrounds, nationalities, and cultures. Getting everyone in the same room (or in the same Skype call) at the same time may already be a real challenge. On top of that, the way in which people communicate and are used to interacting during meetings can vary significantly. A fruitful meeting therefore critically depends on a succinct agenda and strong chairperson.

*“Together with RSG-Japan, RSG-Singapore, and RSG-Taiwan, we organized an international conference series for young researchers and students who are conducting their research in Asia—the Asian Young Research Conference on Computational Biology and Omics Biology (AYRCOB). Organizing this event with such a diverse group of students from more than four different countries was a real challenge and, at the same time, a rewarding learning experience.”*—Joshua SungWoo Yang (president of RSG-Korea, 2007 to 2009)

## Leadership Skills

When you take on the responsibility of leading one of the RSGs, you will soon find out that leadership skills are something very different than management or organizational skills. As a leader, you don't tell people what to do; instead, you will need to coach your team to do what is necessary and motivate your people to walk the extra mile to secure a good project outcome. For many of us, this is the first situation in which true leadership is required. Taking the lead in one of our RSGs thus provides an invaluable experience. Despite the fact that effective leadership is essential for becoming an independent researcher, educational programs hardly ever spend a significant amount of time on the subject.

*“A classic mistake is to allow yourself the attitude ‘let me do it because then it will be done right.’ The most important reason this is wrong is that building a team of people that enjoy working together requires giving people responsibility. By the way, you will be surprised how often the other way is just as good, if not better, than your way.”*—Jeroen de Ridder

Creating the collective responsibility required to be successful as a team is particularly challenging in an organization of volunteers that cannot rely on the leverage of a paycheck or layoff. The solution is to use a leadership style that is built on positive reinforcement and to keep emphasizing the benefits of actively participating in the organization. In our experience and without exception, at the end of the day people will appreciate the new skills obtained while contributing to RSG or ISCB-SC activities.

*“In the ISCB-RSG quiz competition organized by RSG-Western Africa at the Covenant University, Nigeria, I was able to learn the soft skill of conflict negotiation and conflict resolution. We had to resolve a situation in which multiple teams tied for the same position. We solved the issue to everyone's satisfaction by creating several additional questions on the fly to ensure a winner could be appointed.”*—O. Oluwagbemi (webmaster, RSG-Western Africa)

## Networking Skills

Networking is a skill you can almost not escape improving when organizing activities or helping out with events. On frequent occasions, you get a chance to interact with some of the most prominent researchers in the field, for instance, by inviting them to be a speaker at your symposium or a tutor for your workshop. More importantly, however, you build friendships with your RSG and ISCB-SC team members. It is good to realize that your current peers will be the scientific leaders of the future and may be your future collaborators or friendly referees.

*“It is the task of any active volunteer to build and maintain a rich personal network. This is a skill that comes naturally with time; every new event is a chance to meet new people, every conference is a chance to make new friends. I am confident that the people I have met will be useful in my future, but above all, it was always a great and fun experience to meet them and discuss science.”*—Pieter Meysman

However, one could argue that the most important reason to invest in a large network is that it guarantees pleasant computational biology meetings. After all, there is no need to wander around alone or go out for drinks all by yourself!

## Troubleshooting Skills

Running and participating in an RSG is not always without its difficulties; there will always be problems (better thought of as challenges) within an organization. It is confronting these challenges and reflecting on the outcome that teaches us the most valuable lessons and invaluable skills.

The most universal problem for volunteers is that when life or research crops up, it is easy to start postponing that which cannot be postponed. When you are in charge (and in fact, even when you are not), it is very important you take notice if any of your colleagues is struggling with this. This enables you to motivate the person in question to deliver what they promised. It goes without saying that this requires a lot of tact and does not always work. Having a backup plan and people to step in in case someone is unavailable for a while is therefore essential.

## Foresight Skills

Successfully running an RSG not only requires taking care of day-to-day business, it also requires planning ahead to ensure the continuity of the organization in the years to come. Most importantly, a steady supply of new volunteers is essential to the life span of every RSG. This calls for reaching out to new students who might not even have heard of your organization. Once you have attracted interested volunteers, a sound effort to transition responsibilities to the newcomers is really going to pay off.

Having said all this, the hardest part of all for RSG leaders is knowing when to pass the torch to the next person. It is often too easy to stick around for just one more year, because it is just so much fun to be part of a thriving organization of scientists [2].

## Concluding Remarks

From the above, it should be clear that actively participating in the leadership of an RSG will take you a long way in developing the necessary skills to further your career in science. To refine your skillset even further and/or obtain a more theoretical foundation, it may be worth considering that many research or graduate schools offer intensive workshops on soft skills as well. Evidently, after taking such a course, participating in the leadership of an RSG will provide an excellent opportunity to bring the theory into practice.

**About the Authors**

The authors have worked on many aspects of the ISCB Student Council and the Regional Student Group program.

**Jeroen de Ridder** was cofounder of RSG-Netherlands and served as chair for the ISCB Student Council from January 2010 to January 2011.

**Pieter Meysman** was cofounder of RSG-Belgium and served as its president from 2011 to 2012.

**Olugbenga Oluwagbemi** was webmaster (2010–2013) and serves as vice president (2013–present) of RSG-Western Africa

**Thomas Abeel** was cofounder, president (2009), and secretary (2010–2011) of RSG-Europe and served as student representative to the ISCB Board of Directors from January 2011 to January 2013.
